# Histone methyltransferase SETD2: An epigenetic driver in clear cell renal cell carcinoma

**DOI:** 10.3389/fonc.2023.1114461

**Published:** 2023-03-21

**Authors:** Mengxue Yu, Kaiyu Qian, Gang Wang, Yu Xiao, Yuan Zhu, Lingao Ju

**Affiliations:** ^1^Department of Biological Repositories, Zhongnan Hospital of Wuhan University, Wuhan, China; ^2^Department of Urology, Zhongnan Hospital of Wuhan University, Wuhan, China; ^3^Human Genetic Resources Preservation Center of Hubei Province, Wuhan, China; ^4^Wuhan Research Center for Infectious Diseases and Cancer, Chinese Academy of Medical Sciences, Wuhan, China; ^5^Medical Research Institute, Wuhan University, Wuhan, China

**Keywords:** SETD2, clear cell renal cell carcinoma (ccRCC), H3K36me3, epigenetic regulation, mutation

## Abstract

SET domain-containing 2 (SETD2) is a lysine methyltransferase that catalyzes histone H3 lysine36 trimethylation (H3K36me3) and has been revealed to play important roles in the regulation of transcriptional elongation, RNA splicing, and DNA damage repair. *SETD2* mutations have been documented in several cancers, including clear cell renal cell carcinoma (ccRCC). *SETD2* deficiency is associated with cancer occurrence and progression by regulating autophagy flux, general metabolic activity, and replication fork speed. Therefore, SETD2 is considered a potential epigenetic therapeutic target and is the subject of ongoing research on cancer-related diagnosis and treatment. This review presents an overview of the molecular functions of SETD2 in H3K36me3 regulation and its relationship with ccRCC, providing a theoretical basis for subsequent antitumor therapy based on SETD2 or H3K36me3 targets.

## 1 Introduction

Renal cell carcinoma (RCC) is one of the most prevalent malignancies with a case-fatality rate among urinary tract tumors (1, 2). There are several pathological types of renal cancer, such as clear cell RCC (ccRCC), papillary RCC (pRCC), and chromophobe RCC (chRCC). In the WHO classification, with a list of RCC defined molecularly, including TFE3-rearranged RCC, TFEB-rearranged RCC, ELOC (TCEB1)-mutated RCC, Fumarate hydratase (FH)-deficient RCC, Succinate dehydrogenase (SDH)-deficient RCC, ALK-rearranged RCC, SMARCB1-deficient RCC, and so on (3), a molecular perspective to define RCC is necessary. ccRCC is the major type with a high incidence rate and poor prognosis. Remarkably, several secondary mutations of tumor suppressor genes and chromatin regulators have been identified near von Hippel-Lindau (*VHL*), including *PBRM1*, *BAP1*, and *SETD2* (4). Furthermore, metastatic ccRCC occurs in about 30% of patients, and there are few effective treatment options available (5). Despite advances in chemotherapeutic drugs, chemotherapy resistance remains a problem in ccRCC treatment; therefore, there is an urgent need to understand the regulatory mechanism underlying the recurrence and metastasis of ccRCC, identify possible therapeutic targets and develop new therapeutic options.

Epigenetic regulation, including histone modification, plays a crucial role in maintaining eukaryotic genome stability, gene expression regulation, and chromatin structure. Histone H3 lysine 36 trimethylation (H3K36me3) is involved in the regulation of transcriptional activation and RNA splicing, as well as DNA repair and recombination (6). In mammalian cells, SETD2 is the main H3K36me3 methyltransferase (7), and genomic profiling of ccRCC clinical samples revealed high-frequency *SETD2* mutations. SETD2 has been reported to accelerate ccRCC progression (4, 8) and is a potential prognostic and predictive marker in both localized and metastatic RCC (9). This paper reviews the multiple roles and functions of SETD2 in the occurrence and progression of ccRCC.

## 2 Protein structure of SETD2

**Figure 1:**
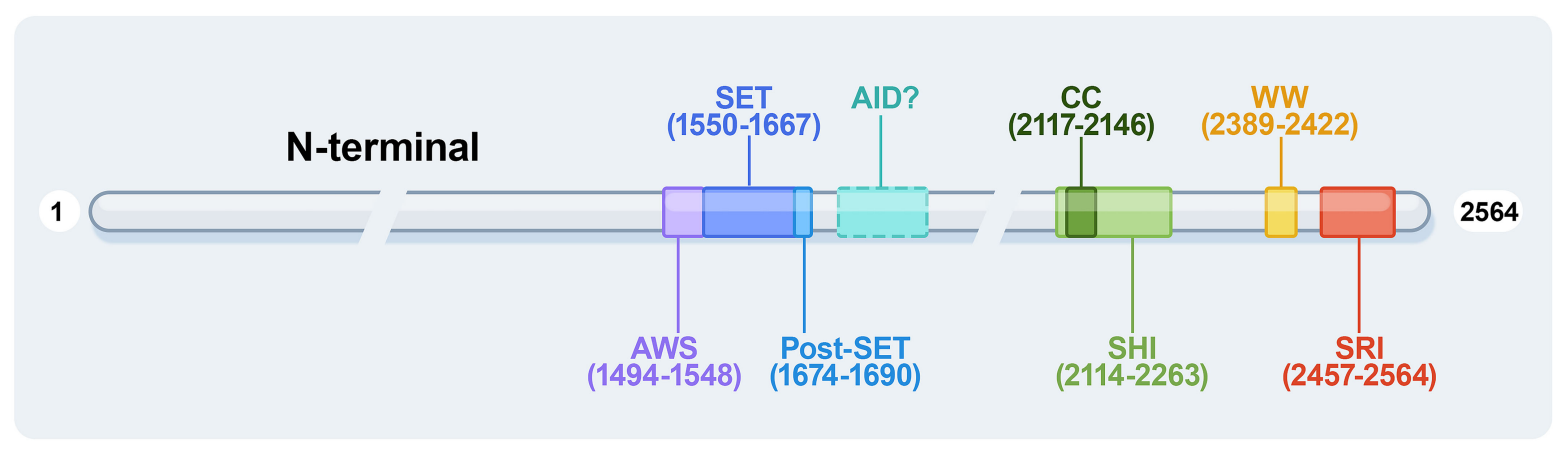
The protein domains of human SETD2. AWS, associated with-SET domain; AID, auto-inhibitory domain; WW, tryptophan-tryptophan domain; CC, coiled-coil domain; SHI, SETD2-hnRNP interaction domain; SRI, Set2-Rpb1 interacting domain.

The human SETD2 gene is located in the p21.31 region of chromosome 3, where the copy number is frequently lost in many tumors. Thus, SETD2 is generally considered a tumor suppressor. The human SETD2 protein consists of several conserved functional domains, containing the AWS (associated with SET)-SET-PS (post-SET) domains, WW domains, SRI (Set2-Rpb1 Interacting domain), SETD2-hnRNP interaction (SHI) domains, and a large unstructured N-terminal domain (**Figure 1**).

### 2.1 The AWS-SET-PS domains

The AWS-SET-PS domains are essential as a catalytic methyltransferase domain for H3K36me3; the AWS and post-SET domains are flanked onto the SET domain at the N- and C-terminally, respectively. All methylation of H3K36me2 to H3K36me3 depends on the SET domain, with S-adenosylmethionine (SAM) as the cofactor, providing an additional methyl (10). It is reported that the H3K36M oncohistone mutation inhibits SETD2 methyltransferase activity; the structure of the SETD2-H3K36M-SAM complex suggests that SAM indirectly affects the SETD2-H3K36M interaction and maintains the SET domain in the proper fold state (11). The AWS-SET-PS domains of SETD2 recognize the α-N helix of histone H3 and bind to the nucleosome DNA by cryo-EM analyses (12).

### 2.2 The Set2-Rpb1 interacting domain

**Figure 2:**
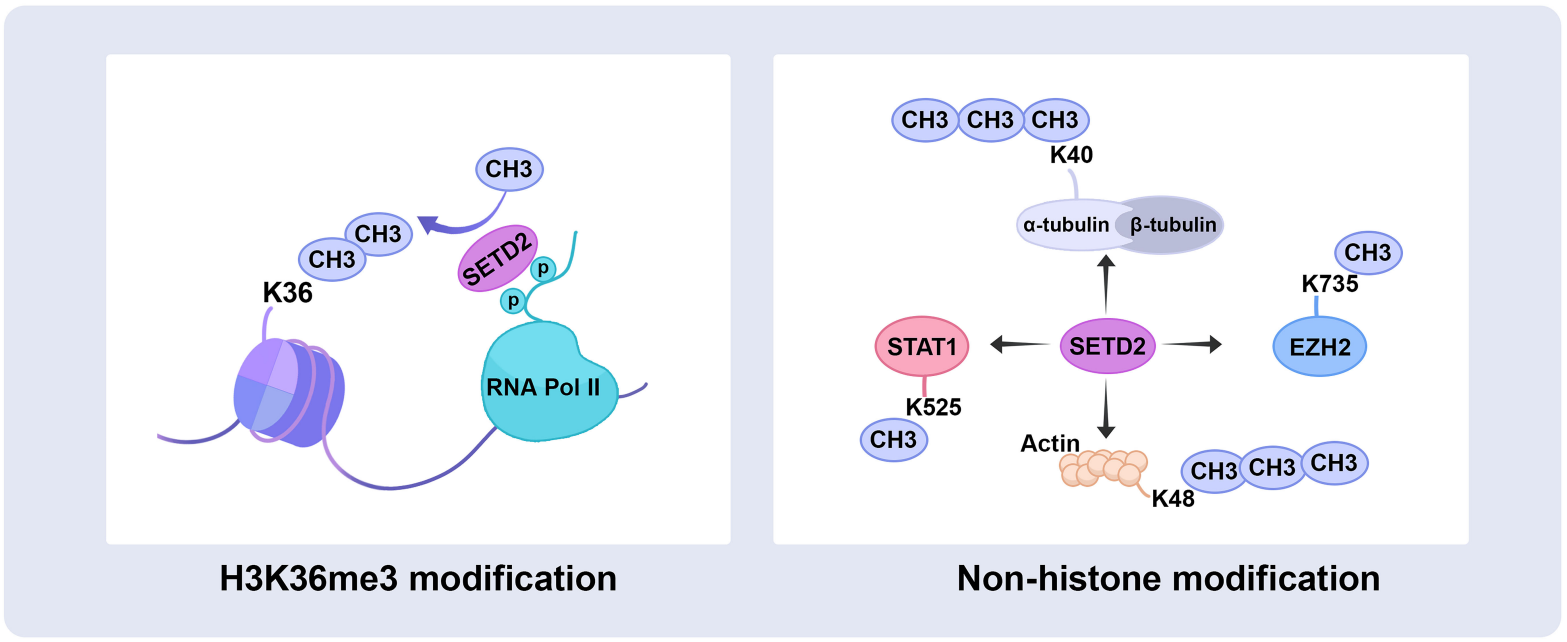
SETD2 catalyzes histone and non-histone substrate methylation. SETD2 has initially identified a methyltransferase that trimethylates H3 histones on lysine 36, also occurs on the non‐histone substrate, trimethylates α-tubulin at lysine 40 and actin at lysine 68, as well as methylates STAT1 at lysine 525 and EZH2 at lysine 735. RNA Pol II, RNA polymerase II; STAT1, signal transducer and activator of transcription 1; EZH2, enhancer of zeste homolog 2.

The SRI domain of 108 amino acids at the C-terminal end is the main region that interacts with RNA polymerase II (RNAPII), entering a transcription elongation phase. The SRI domain binds to RNAPII-C terminal domain (RNAPII-CTD) Ser5P and Ser2P (13) and promotes SETD2 activity to modify H3K36me3, particularly along the 3’ end of the coding sequences of long genes (**Figure 2**). This association is crucial for SETD2 activity and stability. In addition, the SRI domain of SETD2 is also required for microtubule lysine 40 trimethylation (α-TubK40me3) (14, 15) (**Figure 2**). Molenaar et al. recently reported that overexpression of the SRI domain significantly inhibited H3K36me3 and enlarged cell size (16).

### 2.3 The WW domain

The WW domain comprises two conserved tryptophan (W) residues in the SETD2 C-terminus. The WW domain interacts with proline-rich or proline-containing motifs of other proteins mediating protein-protein interactions (17). For example, the WW domain interacts with the Huntingtin (HTT) proline-rich region (PRR) and the actin-binding adaptor HTT-interacting protein 1-related protein (HIP1R), leading to SETD2 tri-methylating actin at lysine 68 (ActK68me3) (**Figure 2**). The SETD2-HTT-HIP1R axis modifies actin, which increases actin polymerization and promotes cell migration (18).

### 2.4 The SHI domain

The structure of the coiled-coil (CC) domain has been predicted by in-silico calculations to be a conserved motif that participates in protein-protein interactions in yeast and promotes protein homodimerization. In human SETD2, the predicted structure of the CC domain is much shorter (19). The SHI domain contains the CC domain and adjacent unstructured sequences in a recently identified region. The histone mark H3K36me3 is known to regulate splicing (8). The SHI region interacts with heterogeneous nuclear ribonucleoprotein L (hnRNP L), RNA-recognition motif 2 (RRM2), as well as other splicing-related factors associated with RNA processing (20). Crystallographic analysis revealed that the Leu-Leu in the SHI domain is important for the interaction (21). Moreover, the double mutant that lacks both the SHI and SRI domains lost practically all catalyzing H3K36me3 activity, indicating that these domains are regulators of SETD2 activity. SETD2 activity toward H3K36me3 modification is similarly influenced by the SHI domain (20).

### 2.5 Auto-inhibitory domain

The AID domain in the middle region of Set2 (a yeast ortholog of human SETD2) suppresses SET domain activity, and the AID domain suppresses its catalytic activity when the SRI domain is lost. AID mutations usually lead to excessive activity of Set2 *in vivo* and promote the abnormal methylation of Set2 to histones (22). The AID and SRI domains synergistically control the catalytic SET domain, with AID mutations resulting in changes in Set2 protein stability and binding to RNAPII-CTD and variable H3K36me3 levels. In summary, Set2 AID exerts repressive effects requiring the presence of the SRI domain and Set2 SRI to interact with RNAPII and histones, ensuring that H3K36 methylation occurs explicitly on the active transcript chromatin. Therefore, under specific growth conditions, the Set2 autoinhibitory domain may serve as a target for other regulators (23). It would be intriguing to ascertain whether the Set2 AID interacts with any proteins and whether this interaction infuses Set2 activity *via* the AID-SRI axis (19). All the above studies are implemented in yeast, but there are no reports about the structure and function of human AID as yet.

### 2.6 The large unstructured N−terminal domain

Human SETD2 has an extended N-terminal region with unknown function (~1400 amino acids) and is unstructured. SETD2 is an unstable protein that depends on the degradation of the proteasome (24). It was recently reported that the N-terminal region regulates its half-life by the proteasome system, and removal of the N-terminal region leads to SETD2 stabilization (25), and a segment (aa 1104-1403) of the N-terminal region contributes to SETD2 degradation by the proteasome (24). SETD2 is an intrinsically aggregation-prone protein, and the N-terminal region contributes to SETD2 droplet formation *in vivo*, a property that is enhanced by its reduced degradation. The N-terminal region is conducive to the liquid-liquid phase separation of the protein, and the phase separation behavior of SETD2 intensifies with the removal of the N-terminal fragment (26). Thus, the N-terminal fragment of SETD2 regulates the amount of SETD2 protein required in the cell and may contribute to its role in regulating transcription and splicing.

## 3 SETD2 and clear cell renal cell carcinomas

### 3.1 *SETD2* mutation and ccRCC

*VHL* inactivation occurs in 90% of all ccRCCs, and several mutations in tumor suppressor genes on chromosome 3p have been identified: *PBRM1*, *BAP1*, and *SETD2* (4, 27). *SETD2* mutations occur in about 15% of ccRCC (4). Mono-allelic and bi-allelic mutations in *SETD2* are observed in many cancers, including ccRCC (28–30). Bi-allelic mutations in *SETD2* cause loss of H3K36me3 in ccRCC (31). *SETD2* gene inactivation mutations are a prevalent molecular feature, and *SETD2* deficiency is associated with ccRCC recurrence and poor prognosis (**Tables 1**, **2**). Moreover, *SETD2* mutations are more frequently found in late-stage ccRCC tumors, which is related to a higher and earlier risk of relapse and poor survival outcomes (9, 50).

**Table 1:** List of *SETD2* mutations reported in ccRCC.

Site	Mutation type	Domain	Function	Ref.
R1625C, R1625H	Missense Mutation	SET	Oncogenic, inactivate SETD2 enzymatic activity	(31–33)
X2413_splice	Splice Site	WW	Oncogenic	(32, 34, 35)
X2478_splice	Splice Site	SRI	Oncogenic, lose the interaction with RNA polymerase II	(32, 34–36)
K2545*	Frame Shift Ins	SRI	Oncogenic, lose the interaction with RNA polymerase II	(32, 34, 35)
T2540Sfs*22, D2504*	Frame Shift Del	SRI	Oncogenic, lose the interaction with RNA polymerase II	(32, 34, 35)
K2511=	Splice Region	SRI	Oncogenic, lose the interaction with RNA polymerase II	(32, 34, 35)
Y2489*	Nonsense Mutation	SRI	Oncogenic, lose the interaction with RNA polymerase II	(32, 34, 35)
X2477_splice	Splice Site	SRI	Oncogenic, lose the interaction with RNA polymerase II	(32, 34, 35)
X2475_splice	Splice Site	SRI	Oncogenic, lose the interaction with RNA polymerase II	(32, 34, 35, 37)
Q2207*	Nonsense Mutation	SHI	Oncogenic	(32, 34, 35)
Y1666H	Missense Mutation	SET	Oncogenic, SETD2 Y1666 interact with H3K36M	(38)
Y1666*	Nonsense Mutation	SET	Oncogenic	(32, 34, 35)
X1572_splice	Splice Site	SET	Oncogenic	(32, 34, 35)
V1656Efs*11	Frame Shift Ins	SET	Oncogenic	(32, 34, 35)
X1640_splice	Splice Site	SET	Oncogenic	(32, 34, 35)
X1672_splice	Splice Region	SET	Oncogenic	(32, 34, 35)
Y1688_L1689delins*	Nonsense Mutation	Post-SET	Oncogenic	(32, 34, 35)
L2124*	Nonsense Mutation	CC	Oncogenic	(32, 34, 35)
X1529_splice	Splice Site	AWS	Oncogenic	(32, 34, 35)
S203Ifs*33, K1969Nfs*2, P1973Lfs*33, K941Rfs*41, T2372Sfs*54, S708Hfs*54, S595Kfs*3, Y1286Sfs*12, L1778Cfs*9, R1994Nfs*9, P1822Qfs*16 (Germline), R1694Sfs*17, L2364Cfs*8, K1863Sfs*2 (Germline), I669*, D289Mfs*12, P1873Nfs*10, D2004Ifs*2, I1194Yfs*42, Y2296Lfs*72, P2380Tfs*31	Frame Shift Del	–	Oncogenic	(32, 34, 35)
Q109*, S185*, Q256*, R368*, R400*, K466*, E505*, K528*, G538*, S543*, Y545*, S560*, S618*, C727*, E777*, R973*, S996*, Y1113*, W1217*, R1322*, Q1368*, Y1472*, R1492*, E1720*, L1748*, W1782*, E1964*, Q2277*	Nonsense Mutation	–	Oncogenic	(32, 34, 35)
S2382Lfs*47, S546Ffs*2, D1456Gfs*28, T2443Nfs*3, P2288Ifs*22, P230Tfs*7 (Germline)	Frame Shift Ins	–	Oncogenic	(32, 34, 35)
X1485_splice, X2450_splice, X2037_splice	Splice Site	–	Oncogenic	(32, 34, 35)
X2037_splice	Splice Region	–	Oncogenic	(32, 34, 35)
R2510H	Missense Mutation	SRI	Globally restore H3K36me3; loss of both tubulin binding and methylation	(15, 31)
G1681fs, Q320fs	Frame Shift Del	SET	Reduce SETD2 enzymatic activity	(39)
R2510L	Missense Mutation	SRI	Reduce SETD2 enzymatic activity	(39)
E978*, Q1409*	Nonsense Mutation	–	Inactivate SETD2 enzymatic activity	(39)
N1734D, S1769P	Missense Mutation	–	Facilitate localization of hMSH6 (hMutSα) to chromatin	(40)
R2132fsX13	Frame Shift Del	–	Result in a PTC 42 nucleotides downstream	(41)
D1616N	Missense Mutation	SET	Influence methyltransferase activity of SETD2	(41)
T2354A	Missense Mutation	–	Affect transcriptional activation activity	(41)
K2541fs	Frame Shift Ins	SRI	Oncogenic, lose the interaction with RNA polymerase II	(37)
E2120fs	Frame Shift Del	CC	Unknown	(37)
F1651Lfs*12	Frame Shift Del	SET	Unknown	(42)
Q2131*	Nonsense Mutation	CC	Unknown	(42)
E2128*	Nonsense Mutation	SHI	Unknown	(42)
T2513I	Missense Mutation	SRI	Unknown	(42)
W2417Lfs*7	Frame Shift Del	WW	Unknown	(42)
C1516S	Missense Mutation	AWS	Unknown	(42)
SETD2-QRICH1	Fusion	–	Oncogenic	(32, 34, 35)

Frame Shift Ins, Frame Shift Insertion; Frame Shift Del, Frame Shift Deletion; PTC, Premature Termination Codons. The asterisk (*) indicates the stop codon.

**Table 2:** Effects and mechanisms of *SETD2* deficiency in ccRCC.

Effect	Mechanism	Cell type	Ref.
Decreased autophagic flux	Increase ATG12 short isoform	ACHN, Caki-1	(43)
	Inhibit the actin-WHAMM interaction	786-O	(44)
Metabolic alterations	Enhance oxidative phosphorylation	786-O	(45)
	Inhibit multiple metabolic-related genes	293T	(46)
Promotes metastases	Induce the recruitment of histone chaperone ASF1A/B and SPT16, increase MMP1 chromatin accessibility	JHRCC12, Caki-2	(47)
Cell cycle arrest	RRM2 expression reduction, dNTP depletion, S-phase arrest	A498	(48)
PKD conversion to ccRCC	Activate the Wnt/β-catenin signaling pathway	PETC, 293T	(49)

ATG12, autophagy-related gene 12; WHAMM, WAS Protein Homolog Associated with Actin, Golgi Membranes, and Microtubules; ASF1A/B, anti-silencing function 1 A/B; SPT16, suppressor of Ty 16; MMP1, matrix metalloproteinase-1; RRM2, Ribonucleotide reductase (RNR) small subunit M2; PKD, Polycystic Kidney Disease.

Referenced by cBioPortal database and reported research (31, 32, 34, 36–38, 42), *SETD2* mutations were identified in ccRCC predominantly inactivating, containing nonsense mutations, missense mutations, frame shift, and fusion, which lead to loss of function, such as mutations R1625C or R1625G, resulting in a complete loss of SETD2 enzymatic activity (31, 33) (**Table 1**). The presence of intratumor heterogeneity was confirmed in metastatic renal-cell carcinoma tumors, which demonstrated independent and different *SETD2* mutations in different sections of an individual tumor (51). Thus, SETD2 plays a critical role in the development and progression of ccRCC.

### 3.2 SETD2 serves as a tumor-suppressor gene in ccRCC

#### 3.2.1 Cryptic transcription

**Figure 3:**
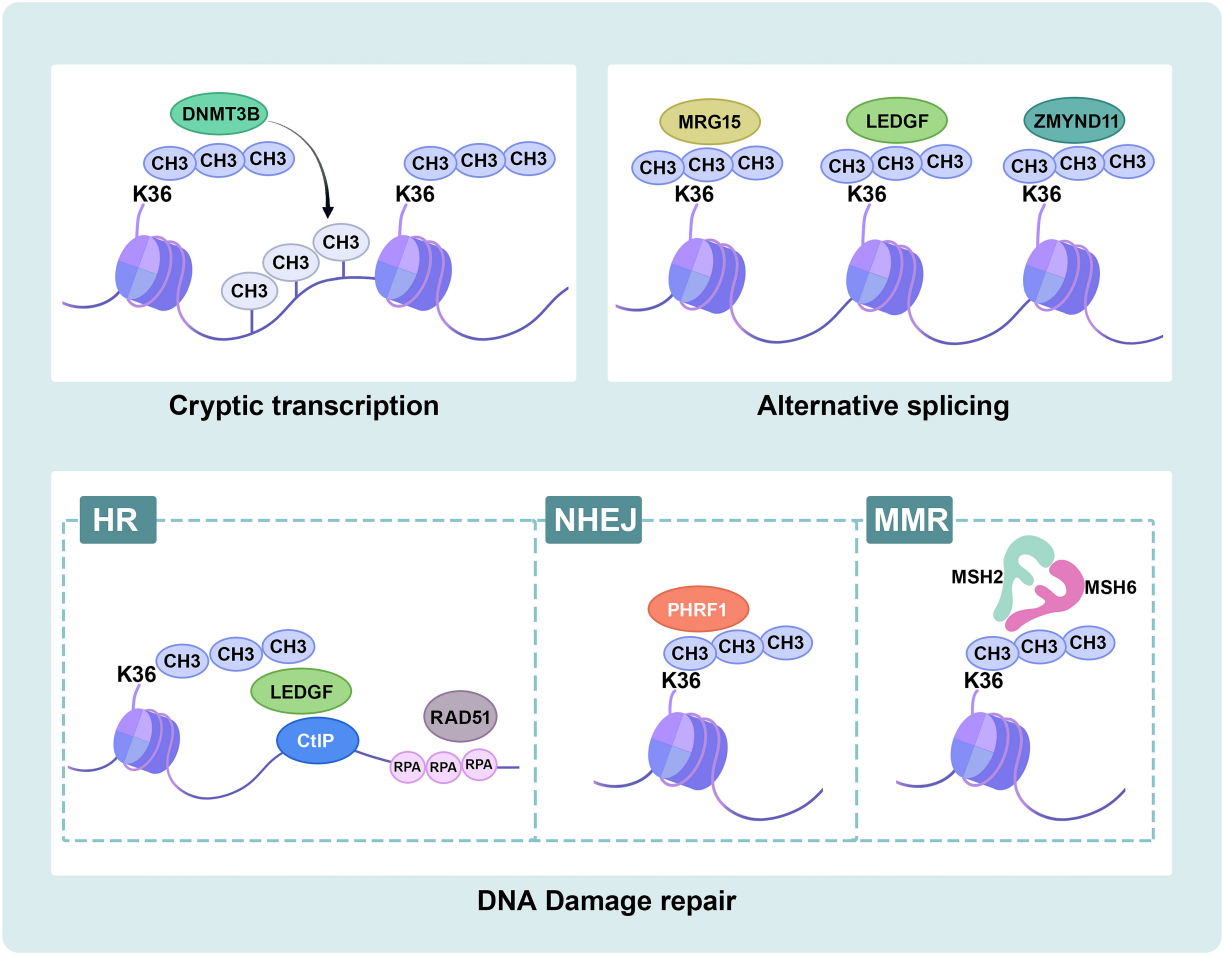
Schematic overview of SETD2 functions. SETD2-mediated H3K36me3 plays important biological roles in ccRCC. Cryptic transcription: SETD2-mediated H3K36me3 recruits DNMT3B to target intragenic DNA methylation. RNA splicing: SETD2-mediated H3K36me3 recruits splice factors MRG15, LEDGF and ZMYND11 to modulate alternative splicing events. DNA damage repair: SETD2-mediated H3K36me3 recruits LEDGF, and LEDGF binds CtIP at the break site to promote HR repair. SETD2-mediated H3K36me3 recruits PHRF1 to modulate NHEJ repair. SETD2-mediated H3K36me3 recruits hMutSα (hMSH2-hMSH6) to MMR repair. DNMT3B, DNA-methyltransferase 3B; MRG15, MORF4-related gene on chromosome 15; LEDGF, lens epithelium-derived growth factor; ZMYND11, Zinc finger MYND-domain containing 11; HR, Homologous recombination; CtIP, C-terminal binding protein interacting protein; RPA, replication protein A; NHEJ, non-homologous end-joining; PHRF1, plant homeodomain of Ring finger domains 1; MMR, mismatch repair; MSH2, MutSα homolog 2; MSH6, MutSα homolog 6.

Cryptic transcription initiates transcription from a downstream “promoter-like” region and produces short and meaningless transcripts in gene bodies. Previous studies have demonstrated that SETD2 suppresses cryptic transcription initiation from within several active gene bodies (52, 53). The histone chaperone FACT and its subunits SPT16 and SPT6 promote transcriptional elongation through nucleosome recombination, and deletion of *SETD2* reduces recruitment to FACT and plays a critical role in repressing cryptic intragenic transcriptional initiation (52). In yeast, Set2-mediated prevention of cryptic intragenic transcription is independent on histone deacetylation (54). In mammalian cells, SETD2-mediated H3K36me3 recruits DNA-methyltransferase 3B (DNMT3B), resulting in a high density of DNA methylation, and thus represses transcription from alternate intragenic promoters or initiation of cryptic transcription (55), protecting RNAPII from inappropriate transcription re-initiation and enforced silence intragenic transcription (53, 56). In conclusion, SETD2 is crucial in maintaining active gene bodies dormant in mammalian cells (**Figure 3**).

#### 3.2.2 RNA splicing

SETD2 is linked to the RNA splicing process. Compared to controls, SHI domain deletion mutation lost interaction with hnRNP L and did not affect splicing events (21). *SETD2*-deficient ccRCC is susceptible to mis-splicing. Gene set enrichment analysis (GSEA) shows that *SETD2*-deficient negatively enriched the gene related to the mRNA splicing pathway (57). A genome-wide transcript profile for *SETD2*-deficient primary ccRCC tumors demonstrated that altered splicing patterns or splicing defects, including intron retention and variation in exon utilization, are widely present in *SETD2*-deficient cancers. Notably, active genes also revealed increased chromatin accessibility (39). The increased chromatin accessibility of upstream abnormally spliced exons and decreased occupancy of nearby nucleosomes significantly contribute to the splicing defect in tumors with H3K36me3 deficiency (58).

Proteins containing the Pro-Trp-Trp-Pro (PWWP) domain play an important role in recognizing H3K36me3. MORF4-related gene on chromosome 15 (MRG15) can bind to H3K36me3 (59, 60) and recruit polypyrimidine tract-binding protein (PTB) to its target alternatively spliced exon sites (61). Lens epithelium-derived growth factor (LEDGF) binds to H3K36me3 (62), as well as to both chromatin and multiple regulators, to modulate alternative splicing events and influence transcription elongation (63, 64). Zinc finger MYND-domain containing 11 (ZMYND11) directly binds to H3K36me3 and H3K36me3-modified chromatin to regulate RNA splicing and Pol II elongation (65, 66). Furthermore, the deficiency of SETD2-mediated H3K36me3 reduces the recruitment of readers, resulting in splicing defects (**Figure 3**).

#### 3.2.3 DNA damage and repair signaling

SETD2 is vital in the DNA damage response (DDR) by generating H3K36me3. Cell death occurs if DNA repair fails, and tumor development may arise from incorrect DNA repair. SETD2 facilitates DNA double-strand breaks (DSBs) repair by homologous recombination (HR), activating replication protein A (RPA) single-stranded DNA (ssDNA)-binding protein complex loading and the formation of RAD51 presynaptic filaments (35, 62, 67, 68). ATM is activated in DSB, then phosphorylates a variety of downstream effector proteins, such as p53. *SETD2*-deficient cancer cells failed to activate p53 and displayed lower cell survival in DNA damage (62, 67, 68). Ectopic expression of demethylase KDM4A decreased H3K36me3 levels and resulted in HR (62). Consistent with this, LEDGF recruits and binds C-terminal binding protein interacting protein (CtIP), promoting HR by CtIP-dependent DNA end resection (69). Accordingly, the loss of SETD2 obstructs HR repair (70, 71). Also, SETD2 promotes DSB repair *via* combination with plant homeodomain (PHD) of Ring finger domains 1 (PHRF1), modulating non-homologous end-joining (NHEJ) and stabilizing genomic integrity (72). SETD2 has also been proven to trigger DNA mismatch repair (MMR). Specifically, the mismatch recognition protein hMutSα (hMSH2-hMSH6), hMSH6 contains a PWWP domain that recruits and interacts with H3K36me3 like many other H3K36me3 effector proteins. hMSH6 foci are reduced in *SETD2* knockdown cancer cells (40). The crystal structure modeling revealed that H3G34R/V mutations block the SETD2 catalytic activity and inhibit H3K36me3-MSH6 interaction from inducing tumorigenesis (73). *SETD2*-deficient cells exhibit microsatellite instability (MSI) with a high frequency of spontaneous mutations (40). Compared to introns and non-transcribed regions, H3K36me3 and MutS are more enriched in exons as well as active transcriptional regions and transcriptionally protect against actively transcribed genes (74). Recent studies suggest that targeting DDR is feasible to achieve immunotherapy in ccRCC (75, 76) (**Figure 3**).

#### 3.2.4 Autophagy

Autophagy is involved in physiological and pathological processes and tightly regulated by a network of autophagy-related genes (ATG). Also, the actin cytoskeleton regulates autophagy dynamics (77). Autophagy is an intracellular degradation system procedure associated with cytoplasmic events, and key epigenetic events are recognized to be significant for this progression. De facto, histone post-translational modification plays a central role in regulating transcriptional programs and epigenetic networks during autophagy (78–83).

Autophagy is an important regulatory process in ccRCC (84–86). The deficiency of *SETD2* in ccRCC cells reduces LC3-II expression, which is linked with abnormal cumulative ATG12 in free and complexes containing ATG12, except for the ATG5-ATG12 complex. Furthermore, *SETD2*-loss deregulates alternative splicing, which is related to increased *ATG12* short isoform and reduced conventional *ATG12* long isoform (43). Another research confirms that *SETD2* knockdown causes a decreased expression of *ATG14* long isoform in HeLa cells (87). Whether *ATG14* long isoforms expression is down-regulated in ccRCC cells with a high-frequency mutation in SETD2 remains to be further investigated.

Autophagy also involves the actin cytoskeleton. As mentioned before, SETD2 trimethylates actin (ActK68me3), cells lacking SETD2 have decreased interaction of the actin nucleation-promoting factor WHAMM with its target actin, actin filaments are required for initiation of autophagy in ccRCC, and autophagy markers LC3-II and p62 are decreased (44).

Recent studies display that the components of the autophagic system play a central role in regulating the innate immune system (88, 89). In pancreatic ductal adenocarcinoma cells, autophagy deficiency results in increased MHC-I expression and increased infiltration of CD8^+^ T cells. Inhibition of autophagy or lysosomal production increases MHC-I expression, enhances the adaptive immune response, and inhibits the generation of tumors (90). Thus, tumor-autonomous autophagy can alter tumor growth by regulating immune responses. SETD2 promotes autophagy flux. Therefore, further understanding the pathways inhibited by *SETD2* deficiency in ccRCC may help identify immunotherapy targets.

#### 3.2.5 Cancer metabolism

ccRCC is considered a metabolic disease and involves several inactivated genes (91), such as *VHL*, controlled tumor energetics and biosynthesis, and the hypoxia pathway (92). The KEGG pathway-based study identified compounds that were present in varied abundance in tumor and normal kidney tissues. Remarkably, most of the upregulated pathways in tumor tissues were engaged in carbohydrate metabolism, whereas the deregulated pathways involved amino acid metabolism (93).

However, the influence of inactivated SETD2 on metabolic reprogramming is unclear. Compared to parental 786-O cells, *SETD2*-deficient cells promote PGC1α, increase oxidative phosphorylation, and elevate mitochondrial oxidative metabolism. Acetyl-CoA is a pivotal substance in biochemical metabolism, which enters the TCA cycle for oxidation and catabolism, and also as a source of fatty acid synthesis, given fatty acid metabolism is always associated with metastasis. Liu et al., hypothesized that enhanced TCA metabolite acetyl-CoA may shunt fatty acid synthesis, resulting in cancer metastasis (45). Compared to wild-type cells, *SETD2* knockout cells inhibit multiple metabolic-related genes in the various metabolic pathways (46). Therefore, tumor metastasis accompanied by metabolic alterations and further metabolic pathways analysis of SETD2 inactivated in ccRCC will have the potential to discover new therapeutics for precision medicine.

#### 3.2.6 Metastases

Previous studies identified an association between SETD2 mutations and the prognosis of patients with localized ccRCC. The mono-allelic mutant of *SETD2* is insignificant in H3K36me3 modification. *SETD2* loss-of-function mutations were revealed in 10%~20% of primary ccRCC tumors, increasing to 30%~60% of metastatic ccRCC tumors. A significant reduction in H3K36 methylation was also found in both ccRCC cell lines and patient samples, suggesting the potential involvement of SETD2 in driving ccRCC metastatic progression (8, 9). In the TCGA cohort, *SETD2* mutations were correlated with poorer cancer-specific survival in ccRCC patients (50). Immunohistochemical staining displayed a gradually decreasing H3K36me3 modification with distant metastases from primary ccRCC tumors. During the progression of ccRCC, H3K36me3 is reduced in distant metastases, and regional H3K36me3 alterations influence alternative splicing in ccRCC (94–97). The H3K36me3 dysregulation axis is linked to an increased risk of death from RCC. Specifically, this connection is substantial, especially for patients with low-risk malignancies (98); however, the mechanism by which SETD2 causes cell metastasis has not been fully elucidated.

The activation of enhancer elements that promote metastatic carcinoma progression has been proven in several cancers, including ccRCC (99–101). Increased chromatin accessibility containing activating enhancers is regulated by aberrant histone chaperone recruitment and activity (102, 103). A recent study has shown that *SETD2* deficiency mediated reduction of H3K36me3 induced the recruitment of histone chaperone ASF1A/B and SPT16, increased MMP1 chromatin accessibility, and activated enhancers to drive genes involved in metastasis, promoted ccRCC metastasis (47).

#### 3.2.7 Cell proliferation and cell cycle regulation

SETD2 stabilization increases cell proliferation contrary to its canonical role as a tumor suppressor (25). According to Li et al., decreased *SETD2* reduces cell proliferation and can be restored by *CDK1* knockdown. Multiple SETD2-regulated cellular pathways suppress cancer development and uncover mechanisms underlying aberrant cell cycle regulation in *SETD2*-depleted cells (46). SETD2 is a tumor suppressor in renal primary tubular epithelial cells (PTECs). The proliferative capacity of SETD2-knockdown PTECs was higher than that of SETD2 wild-type PTECs, indicating that SETD2 inactivation enables PTECs to facilitate a malignant transformation toward ccRCC (67).

Generally, DNA damage could cause cell cycle arrest. The abundance of H3K36me3 ensures the recruitment of DNA damage repair key proteins during DNA replication to restore genome integrity in G1 and early S phase (40, 104). Replication fork speed is also decreased in ccRCC cells when *SETD2* is depleted (35). Throughout the cell cycle, the SETD2 protein level is minimal in G1 and maximal in G2/M. Both H3K36me3 and WEE1 are critical in DNA replication and promote ribonucleotide reductase subunit (RRM2) expression, respectively. In *SETD2*-deficient cells, WEE1 inhibition reduces dNTP and RRM2 with higher sensitivity, resulting in S-phase arrest (48).

In recent studies, Helena et al. and Zhu et al. found SETD2 can also catalyze H3K37me1 and H3K14me3, H3K14me3 recruits the RPA complex to active Ataxia telangiectasia and Rad3 related (ATR) during replication stress, which plays a crucial role in the DNA replication stress response and negatively regulates replication initiation, the deletion of *SETD2* reduces replication stress in the absence of H3K37me1 and H3K14me3 (105, 106). In conclusion, SETD2 controls the proper course of the S-phase, and catalyzes H3K37me1 and H3K14me3 to regulate the replication progress. However, the detailed correlation between SETD2 and cell cycle regulation is still incomplete and requires further exploration.

#### 3.2.8 Non-histone substrates of SETD2

SETD2 is the main H3K36me3 methyltransferase in mammalian cells. Recent studies have suggested that SETD2 could also catalyze non-histone substrate methylation. During ccRCC mitosis, SETD2 trimethylates α-TubK40me3 and maintains genomic stability. Mono-allelic mutation of SETD2 results in α-TubK40me3 deficiency, leading to chromosome abnormalities and genomic instability exhibiting multipolar spindle formation, chromosome bridges, micronuclei, polyploidy, and multiple nuclei (14). SETD2, as a chromatocytoskeletal remodeler, trimethylates ActK68me3. The SETD2-HTT-HIP1R axis modifies actin, which increases actin polymerization and promotes ccRCC migration (18). In addition, SETD2 methylates STAT1 on lysine 525 promotes IFNα-dependent antiviral immunity (107), and methylates EZH2 on lysine 735 inhibits prostate cancer metastasis (33). Since SETD2 and EZH2 commonly occur abnormally in urological cancers, the SETD2-EZH2 axis may also be promising targets for pharmacological intervention in ccRCC. In order to search the specificity substrate sequence of SETD2, the amino acid specificity profile of the SETD2 substrate sequence was determined by the peptide SPOT arrays and find the super-matching methylation site on K666 of FBN-1 (108). Further cytological work is still needed to demonstrate that FBN1 is a methylated substrate of SETD2.

A recent study reported that SETD2 could indirectly methylate non-histone substrates, loss of *SETD2* increases protein translation-related gene expression and decreases eEF1A1 K165me3 and K318me1 in ccRCC, but SETD2 is associated with eEF1A1 methylation indirectly, SET domain of SETD2 regulated the expression of EEF1AKMT2 and EEF1AKMT3, EEF1AKMT3 methylates eEF1A1 on lysine 165 and EEF1AKMT2 methylates eEF1A1 on lysine 318 (109). Finally, the discovery of SETD2 for non-histone substrates is particularly crucial for a more in-depth understanding of its biological role (**Figure 2**).

#### 3.2.9 Other functions

Recent research has depicted that multiple chromatin remodeling enzymes are genetically inactive in ccRCC. Even though there is emerging evidence that epigenetic changes are important in cancer, only DNA methylation changes have been identified (92). Widespread DNA hypomethylation correlates to the mutation of the H3K36 methyltransferase SETD2 (94).

Patients with polycystic kidney disease (PKD) have a high probability of converting to RCC. However, there is a paucity of knowledge regarding how PKD can develop into RCC, necessitating further research into genetic alterations or the regulation of signaling pathways. Li et al. found that SETD2 deletion can lead to increased activation of the Wnt/β-catenin signaling pathway and promote epithelial-mesenchymal transition and tumor formation. SETD2 plays an essential role in the process of the conversion of PKD to ccRCC (49).

Emerging evidence suggests that exosomal circRNAs might be potential cancer biomarkers (110–112). He et al. reported that circulating exosomal mRNA (emRNA) is a potential diagnostic biomarker of ccRCC; thus, an emRNA-based screening signature could be developed to provide noninvasive indicators for ccRCC (113).

## 4 Conclusion

SETD2-mediated H3K36me3 enhances transcriptional elongation and is also involved in DNA damage repair and alternative splicing (**Figures 2**, **3**). *SETD2* mutations have been identified in ccRCC (41), but further research should focus on the association with the function of SETD2 and ccRCC. Loss of SETD2 in ccRCC is related to decreased autophagy processing, greater levels of general metabolic activity, poorer cancer-specific survival in ccRCC patients, and slower replication fork speed.

As a tumor suppressor, *SETD2* may serve as a biomarker to reduce drug resistance to targeted therapy and as a potential therapeutic target to promote individualized treatment and improve patient survival. The TCGA pan-cancer cohort shows that patients with *SETD2* mutations have a higher immune-related gene expression and MSI. Clinical data analysis of cancer patients treated with immune checkpoint inhibitors demonstrated that *SETD2* mutation is a potential biomarker (114). 5-aza-2’-deoxycytidine (DAC) is used clinically to treat tumors with mutations in chromatin regulators, which competitively inhibits DNA methyltransferase activity and demethylates DNA. H3K36me3 is reduced in *SETD2*-deficient tumor cells, decreasing the recruitment of DNMT3B and the methylation of DNA, increasing interferon immune responses and the expression of transposable elements, therefore improving the sensitivity to DAC. In wild-type tumors, the number of myeloid-derived immune suppressive cell (MDSC) increased with DAC treatment. In the SETD2-knockdown tumor model, increased CD8^+^ T cell infiltration and fewer MDSC following combined treatment with DAC and anti-PD-L1. ccRCC with altered *SETD2* gene provides preclinical support for a therapeutic target for DAC and anti-PD-L1 (57). A case report about advanced HCC showed that immunotherapy could be effective, leading to long-term survival, and they focused on two mutated genes, *SETD2* and *LRP1B*, to further explore (115). Thus, the hypermutated *SETD2* in ccRCC is worthy of attention.

With current innovations in genome engineering and proteomics, the role of SETD2 in normal cells and cancer will be better understood at the molecular level. Nonetheless, it is urgent to explore whether and how SETD2 regulates the molecular mechanisms of recurrence and ccRCC metastasis.

Furthermore, SMYD5 and SETD5 were also demonstrated to catalyze H3K36me3 (7, 116). A growing number of enzymes were initially discovered for methylating additional amino acid residues of histones and other proteins (117), so a reanalysis of known histone methyltransferases is necessary.

In conclusion, the in-depth study of SETD2 during tumor formation and development is warranted for diagnosing, treating, and preventing tumors. It is anticipated that further epigenetics studies will reveal the regulatory pathway of SETD2 expression.

## Author contributions

YZ and LJ supervised the review study. MY, YZ, and LJ reviewed the literature and drafted the first draft. GW and KQ provided suggestions to improve the draft. MY, YX, and LJ edited the figures and tables. All authors contributed to the article and approved the submitted version.
